# Voltage dependent anion channel-1 regulates death receptor mediated apoptosis by enabling cleavage of caspase-8

**DOI:** 10.1186/1471-2407-10-380

**Published:** 2010-07-20

**Authors:** Alex D Chacko, Fabio Liberante, Ian Paul, Daniel B Longley, Dean A Fennell

**Affiliations:** 1 Queen's University Belfast, Centre for Cancer Research and Cell Biology, Belfast, UK

## Abstract

**Background:**

Activation of the extrinsic apoptosis pathway by tumour necrosis factor related apoptosis inducing ligand (TRAIL) is a novel therapeutic strategy for treating cancer that is currently under clinical evaluation. Identification of molecular biomarkers of resistance is likely to play an important role in predicting clinical anti tumour activity. The involvement of the mitochondrial type 1 voltage dependent anion channel (VDAC1) in regulating apoptosis has been highly debated. To date, a functional role in regulating the extrinsic apoptosis pathway has not been formally excluded.

**Methods:**

We carried out stable and transient RNAi knockdowns of VDAC1 in non-small cell lung cancer cells, and stimulated the extrinsic apoptotic pathway principally by incubating cells with the death ligand TRAIL. We used in-vitro apoptotic and cell viability assays, as well as western blot for markers of apoptosis, to demonstrate that TRAIL-induced toxicity is VDAC1 dependant. Confocal microscopy and mitochondrial fractionation were used to determine the importance of mitochondria for caspase-8 activation.

**Results:**

Here we show that either stable or transient knockdown of VDAC1 is sufficient to antagonize TRAIL mediated apoptosis in non-small cell lung cancer (NSCLC) cells. Specifically, VDAC1 is required for processing of procaspase-8 to its fully active p18 form at the mitochondria. Loss of VDAC1 does not alter mitochondrial sensitivity to exogenous caspase-8-cleaved BID induced mitochondrial depolarization, even though VDAC1 expression is essential for TRAIL dependent activation of the intrinsic apoptosis pathway. Furthermore, expression of exogenous VDAC1 restores the apoptotic response to TRAIL in cells in which endogenous VDAC1 has been selectively silenced.

**Conclusions:**

Expression of VDAC1 is required for full processing and activation of caspase-8 and supports a role for mitochondria in regulating apoptosis signaling via the death receptor pathway.

## Background

The voltage dependent anion channel 1 (VDAC1) is a conserved beta barreled pore forming protein integral to the outer mitochondrial membrane where it regulates ATP/ADP exchange and respiratory control [1]. The functional role of the VDAC proteins VDAC1, 2, and 3 in the regulation of apoptosis remains controversial. Different VDAC proteins exhibit distinct apoptosis regulating functions as evidenced by the antagonism of BAK-induced apoptosis by VDAC2 [2]. A pro-apoptotic role for VDAC1 has been implicated in some cell death models. For example, knockdown of VDAC1 has been reported to abrogate BAX activation and apoptosis following cisplatin treatment in NSCLC cells [3]. VDAC1 has been shown to be required for endostatin-induced endothelial cell apoptosis [4]. Knockout of all three VDAC isoforms does not indicate a direct role in regulating calcium or BID induced mitochondrial apoptosis [5]. However, hexokinase II binds VDAC1 and this interaction has been implicated in regulating cell survival downstream of AKT [6], and blocking ion transport through VDAC1 following toxic insult has been shown to reduce subsequent apoptosis [7].

The potential involvement of VDAC1 in regulating death receptor mediated apoptosis has not been determined. The extrinsic death pathway involves binding of ligands such as TRAIL [8] or FAS [9] to receptors of the Tumour Necrosis Factor Receptor family. This results in recruitment and activation of initiator caspase-8 at the death-inducing signaling complex (DISC), resulting in cleavage of the 53/55 kDa procaspase to catalytically active p43 and p18 forms [10]. Cleaved caspase-8 then directly activates the executioner caspases 3 and 7 [11], and the mitochondrial apoptosis pathway through cleavage of the 23 kDa BID protein to its truncated form tBID, promoting oligomerisation of BAX and BAK [12,13]. Mitochondrial cardiolipin has been proposed to regulate translocation and activation of caspase-8, implicating this organelle in extrinsic death pathway regulation [14,15], and a number of studies have shown that procaspase-8 and p18-caspase-8 localise to the mitochondria [16-18]. Non-small cell lung cancer (NSCLC) cells express relatively high levels of procaspase-8 and are sensitive to induction of apoptosis by TRAIL compared with normal cells both *in vitro *and *in vivo *[19,20]. Accordingly, there is interest in the potential clinical application of TRAIL, and TRAIL receptor agonists in NSCLC and other tumour types [20,21]. In this study we show that VDAC1 is necessary for full caspase-8 activation and apoptosis following activation of death receptors by TRAIL, FAS or FLIP siRNA knockdown in NSCLC cells, implicating a novel functional role for mitochondria in regulating death ligand induced apoptosis.

## Results

### Knockdown of VDAC1 inhibits TRAIL induced apoptosis

**Figure 1:**
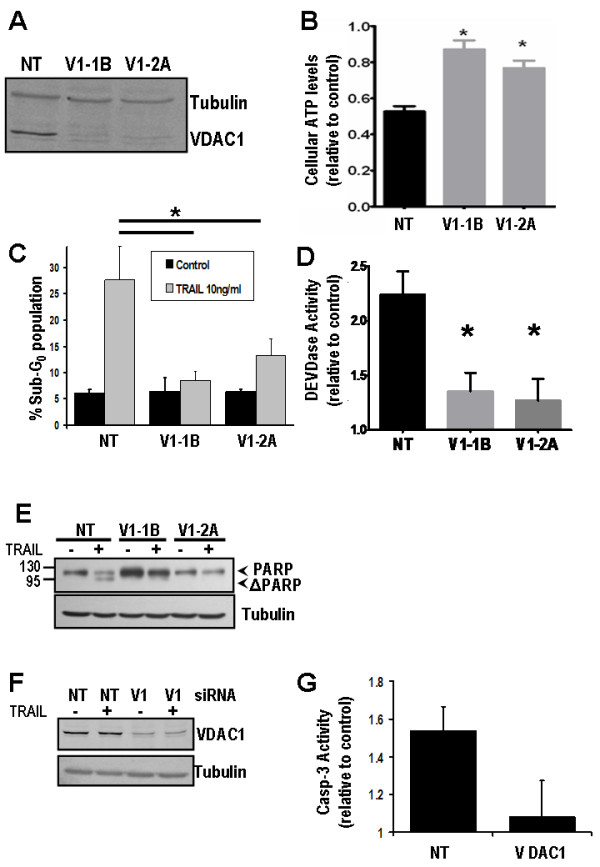
**VDAC1 silencing blocks TRAIL induced apoptosis**. **(A) **Western blot showing H460 clones with stable knockdown of VDAC1 by different shRNA sequences (V1-1B and V1-2A), and control cells expressing non-targeting shRNA (NT). **(B) **ATPase viability assay showing reduction in viability 24 h following 10 ng/ml TRAIL treatment, which is significantly attenuated in VDAC1 knockdown clones compared to NT control cells (*p < 0.0001 v sh-NT). **(C) **Flow cytometry of PI-stained H460 clones following TRAIL treatment. Apoptotic cells are indicated by increase in sub-G_0_/G_1 _population (*p < 0.05). **(D) **Luminescent DEVD-ase assay measures caspase-3-like activity in sh-NT and sh-VDAC1 cells 24 h after TRAIL treatment (10 ng/ml), shown relative to time-match control for each cell line. Increase in caspase-3 activity in NT control is significantly attenuated in sh-VDAC1 cells (*p < 0.0005 v sh-NT). **(E) **Western blot for PARP in sh-NT and sh-VDAC1 cells following TRAIL treatment. PARP cleavage occurs in sh-NT control cells but not sh-VDAC1-1B or sh-VDAC1-2A cells. **(F) **Western blot of H460 cells transiently transfected with siRNA to VDAC1 (V1) or Non-Targeting siRNA (NT). **(G) **Transient siRNA knockdown of VDAC1 was carried out and cells were treated with TRAIL. DEVD-ase assay showing significant attenuation of caspase-3-like activity following VDAC1 knockdown.

To examine the potential role of VDAC1 in regulating extrinsic pathway death signaling in NSCLC cells, we created H460 clones, stably expressing shRNA targeted to VDAC1. Two stable clones with different VDAC1 targeting sequences were generated and compared with non-targeting shRNA (sh-NT). VDAC1 protein expression in clones V1-1B and V1-2A was reduced to almost undetectable levels by Western blot (Figure 1A). Following exposure to IC_50 _concentration of TRAIL ligand, sh-NT control cells reduced their viability; however this did not occur in sh-VDAC1 cells (Figure 1B). Induction of apoptosis evidenced by sub-G_0_/G_1 _fraction was inhibited by VDAC1 silencing (Figure 1C), as was TRAIL-induced activation of caspase-3 (Figure 1D). Cleavage of poly-ADP ribose polymerase (PARP) to its truncated form (ΔPARP) was observed in TRAIL treated sh-NT clones, but not in sh-VDAC1 clones (Figure 1E). As confirmation of these findings, transient knockdown of VDAC1 using a different siRNA sequence (Figure 1F) also prevented caspase-3 activation following TRAIL (Figure 1G). These data indicate that knockdown of VDAC1 inhibits TRAIL-induced apoptosis.

### VDAC1 is required for TRAIL induced mitochondrial formation of Caspase-8-p18

**Figure 2:**
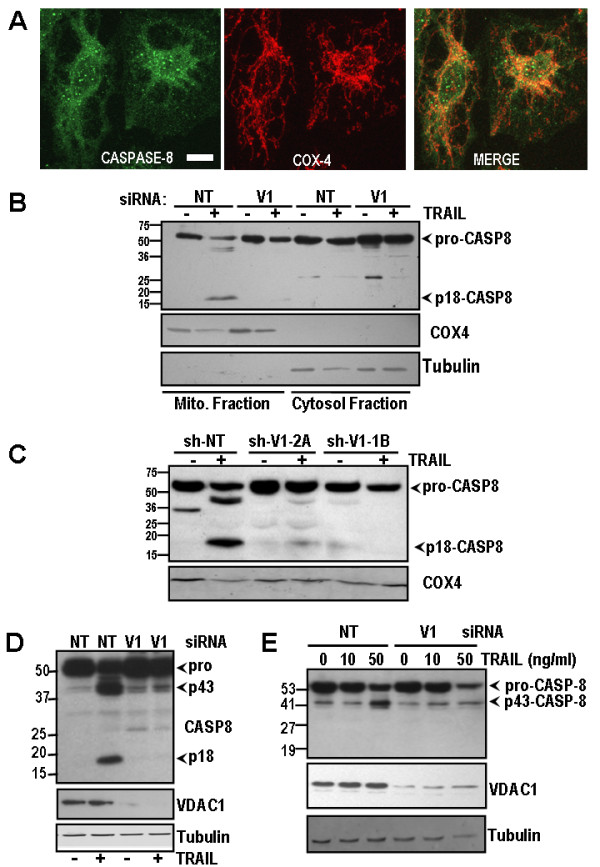
**VDAC1 silencing blocks cleavage of caspase-8 to its p18 subunit**. **(A) **Confocal microscopy image showing colocalisation of Caspase-8 (green) with the mitochondrial marker protein COX-4 (red) in H460 cells. Colocalisation is indicated by yellow staining in merged image. **(B) **Caspase-8 western blot of mitochondrial and cytosolic fractions of cells transiently transfected with NT siRNA or VDAC1 siRNA. Catalytically active caspase-8-p18 appears in NT-siRNA control, but not VDAC1-siRNA cells, following TRAIL. Caspase-8-p18 is predominantly in mitochondrial fraction, but excluded from cyotsolic fraction. COX4 blot is shown as mitochondrial marker; α-tubulin is shown as cytosolic marker. **(C) **Caspase-8 western blot on mitochondrial fraction from H460 shRNA clones treated with 10 ng/ml TRAIL for 24 h. Appearance of 18 kDa form of caspase-8 is strong in mitochondrial fraction of sh-NT control cells but is abolished in sh-VDAC1-1B and sh-VDAC1-2A cell lines. COX4 is shown as mitochondrial loading control. **(D) **Caspase-8 western blot of cell lysates from MOR cell line transfected with NT siRNA or VDAC1 siRNA. Caspase-8-p18 appears in NT-siRNA control, but not VDAC1-siRNA cells, following 10 ng/ml TRAIL treatment. **(E) **Caspase-8 western blot of cell lysates from SKMES cell line transfected with NT siRNA or VDAC1 siRNA. Increase in caspase-8 activation to p43 isoform is detected in NT-siRNA cells following 50 ng/ml TRAIL, but not in VDAC1-siRNA cells. Caspase-8 p18 was not detectable in SKMES cell line at reasonable concentrations of TRAIL.

Caspase-8 localises to mitochondria in various cell lines, where it can undergo further processing and activation [14-18]. Using confocal microscopy, we examined localisation of caspase-8 in H460 cells. Caspase-8 partially colocalised with the mitochondrial marker COX-4 (Figure 2A). Colocalisation was most evident in the perinuclear region, although cytosolic foci of caspase-8 were visible outwith the mitochondrial COX-4 stained area. We therefore examined whether VDAC1 expression affects caspase-8 processing in the mitochondrial compartment. H460 cells were transfected with VDAC1 siRNA or non-targeting (NT) siRNA, incubated with TRAIL for 24 h, and mitochondrial and cytosolic fractions extracted. Activation of caspase-8 involves processing of the 53 kDa pro-caspase zymogen, and intermediate forms, to the catalytically active p18 form. Following TRAIL treatment, activation of caspase-8-p18 protein was observed in the mitochondrial fraction from NT control cells, but this was abolished by VDAC1 silencing (Figure 2B). The active caspase-8-p18 protein was concentrated in the mitochondrial fraction. Cleavage of caspase-8 to p18 was also significantly reduced following TRAIL in the sh-VDAC1 knockdown cells, whereas caspase-8 p18 was strongly detected in the mitochondrial fraction of sh-NT control cells (Figure 2C).

To confirm whether the VDAC1 requirement for caspase-8 activation could be generalised to other cells, VDAC1 was silenced in the MOR NSCLC cell line. Following 10 ng/ml TRAIL, strong accumulation of caspase-8-p18 was observed in NT siRNA treated cells, but this was abolished by knockdown of VDAC1 (Figure 2D). A similar effect was observed in SKMES (Figure 2E), although only p43-caspase-8 was readily detectable in these cells. These results indicate that TRAIL-induced caspase-8 cleavage and apoptosis are VDAC1-dependent.

**Figure 3:**
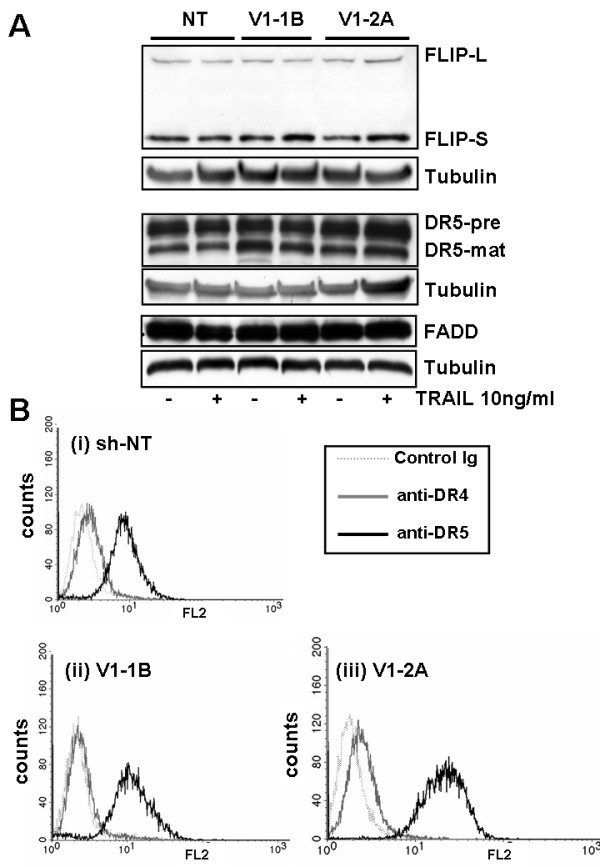
**DISC components are unchanged in VDAC1 knockdown cells**. **(A) **Western blots showing expression of TRAIL receptor DR5, c-FLIP, and FADD, which are of comparable levels in sh-NT control and sh-VDAC1 cells, both before and after TRAIL treatment. **(B) **Flow cytometry showing cell surface expression of TRAIL receptors DR4 and DR5 in sh-NT and sh-VDAC1 cell lines. All cells show high levels of DR5 expression and relatively low levels of DR4.

To exclude alterations in the expression of DISC proteins due to clonal selection in sh-VDAC1 cells, the integrity of key components of the DISC was examined. Expression levels of TRAIL receptor DR5, caspase-8 antagonist c-FLIP, and caspase-8 recruiting protein FADD were all unchanged in sh-VDAC1 clones as compared with sh-NT cells (Figure 3A). No differences in cell surface expression of TRAIL receptor DR5 were detected in the sh-VDAC1 cells compared with sh-NT cells; DR4 was barely detectable in all cells (Figure 3B).

### VDAC1 regulates TRAIL induced BID cleavage and mitochondrial apoptosis

**Figure 4:**
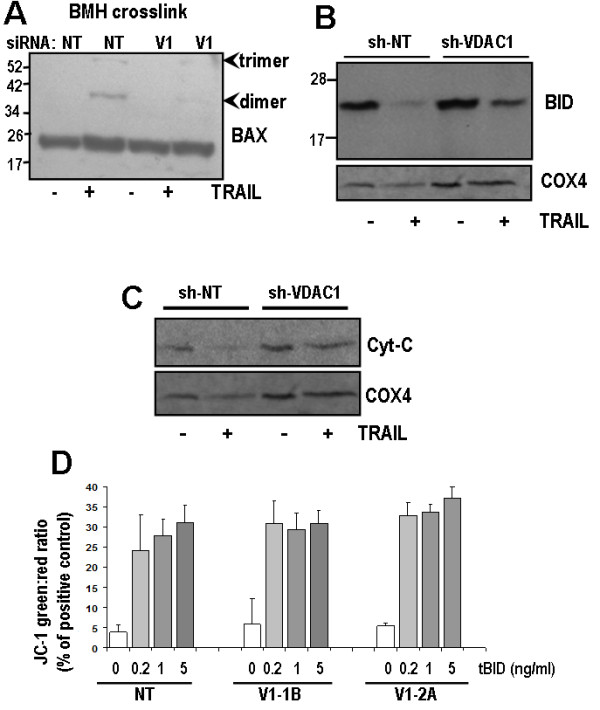
**VDAC1 silencing blocks TRAIL induced mitochondrial apoptosis**. **(A) **Western blot of BAX in BMH-crosslinked mitochondrial fraction from TRAIL-treated or time-matched cells. Dimers and trimers of BAX indicating BAX activation appear in NT cells following TRAIL but this is reduced in VDAC1 knockdown cells. **(B) **Western blot of BID in sh-NT and sh-VDAC1 cells. Full length p23-BID is activated by caspase-8 mediated cleavage in mitochondria following TRAIL treatment in sh-NT control cells but is not processed in the VDAC1 knockdown cells. We were unable to detect the 16 kDa tBID fragment by western blot. COX4 is shown as loading control. **(C) **Western blot of cytochrome-C in mitochondrial fraction of sh-NT and sh-VDAC1-2A cells. Cyt-C is lost from sh-NT control mitochondria following TRAIL, indicating mitochondrial outer membrane permeabilization (MOMP). MOMP is not observed in VDAC1 knockdown cells. **(D) **Mitochondria were extracted from cells shown and were resuspended in respiration buffer and stained with JC-1 dye (see methods) to measure mitochondrial polarization status. 25 μg of mitochondria were aliquoted per well of 96-well plate (n = 5) and incubated with recombinant caspase-8-cleaved BID (tBID) at concentrations shown for 45 minutes. 1% Triton-X treatment was used as positive control for full depolarization. Ratio of green (530 nm) to red (580 nm) fluorescence of JC-1 increases as mitochondria depolarize.

Caspase-8 activates the mitochondrial (intrinsic) apoptosis pathway by cleaving BID to its truncated, active isoform, tBID, which then directly activates BAX and BAK [12,13]. Inhibition of caspase-8 should therefore prevent mitochondrial BAX/BAK activation by TRAIL. To test this hypothesis TRAIL-induced BAX oligomerization was measured by cross-linking of mitochondria with BMH. This was not observed in the sh-VDAC1 clones compared with sh-NT clones in which dimeric and trimeric oligomers of BAX were clearly formed (Figure 4A). Processing of BID was markedly reduced in sh-VDAC1 cells following TRAIL treatment compared to sh-NT clones (Figure 4B). Mitochondrial outer membrane permeabilization evidenced by release of cytochrome C was observed following TRAIL in sh-NT cells, but not in sh-VDAC1 cells (Figure 4C). The lack of intrinsic pathway activation following TRAIL in VDAC1 silenced cells could be due to impairment of BAX/BAK activation, if they are regulated by VDAC1. To test this hypothesis, mitochondria isolated from sh-NT control cells and sh-VDAC1 knockdown cells were incubated with recombinant caspase-8-cleaved BID (tBID). Mitochondrial depolarisation induced by tBID was not inhibited by VDAC1 silencing (Figure 4D). Taken together, VDAC1 is required for TRAIL-induced caspase-8 processing of BID leading to inhibition of TRAIL-induced BAX activation and cytochrome C release, but does not affect the mitochondrial apoptotic response downstream of tBID.

### VDAC1 is required for caspase-8 activation induced by FAS ligand and FLIP silencing

**Figure 5:**
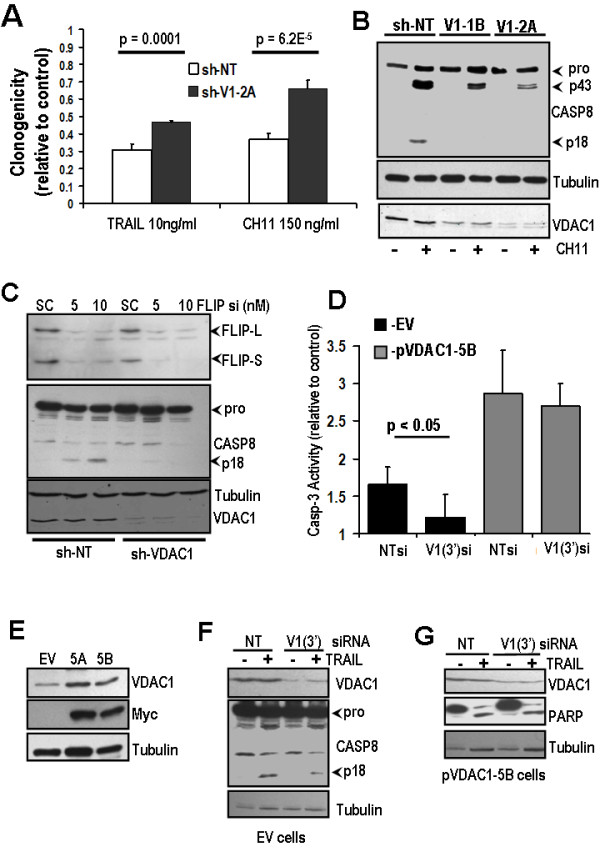
**VDAC1 is required for FAS ligand-induced and FLIP silencing-induced activation of caspase-8**. **(A) **Clonogenic assay shows impairment of cell regrowth in sh-NT and sh-VDAC1 cells following exposure to TRAIL or CH11. Clonogenicity is significantly reduced in sh-VDAC1 cells compared to sh-NT cells for both death ligands. **(B) **Cells were treated with 150 ng/ml FAS ligand CH11. Western blot shows that caspase-8-p18 is generated in sh-NT cells following treatment with CH11, but this is significantly reduced in sh-VDAC1 cells. **(C) **sh-NT or sh-VDAC1 cells were transfected with c-FLIP, or scrambled control siRNA. Caspase-8-p18 was detectable 24 h post FLIP siRNA treatment in sh-NT cells, but not sh-VDAC1 cells. H460 cells were generated that stably expressed the coding region only of VDAC1 fused to myc epitope tag (pVDAC1-5A or pVDAC1-5B), or empty vector (EV). **(D) **DEVDase assay showing increased caspase-3 activity in pVDAC1 cells following 10 ng/ml TRAIL treatment, which is unaffected by V1-3' siRNA, whereas control cells show reduced caspase-3 activity in comparison, which is significantly attenuated by V1-3'siRNA. **(E) **Western blot shows expression of Myc tag and a moderate increase in overall VDAC1 in pVDAC1-5A and pVDAC1-5B. **(F) **siRNA targeting 3'UTR of VDAC1 [V1(3')] causes moderate VDAC1 knockdown in EV cells, but not in pVDAC1 cells. TRAIL treatment shows that V1(3') siRNA reduces processing to Casp-8 (p18) in EV cells. **(G) **Myc-tagged VDAC1 expressing cells exhibit increased sensitivity to TRAIL as shown by increased processing of PARP.

To determine if VDAC1 could regulate caspase-8 activation induced by extrinsic pathway agonists other than TRAIL, cells were treated with FAS ligand (CH11), re-plated, and clonogenicity examined after 7 days. FAS ligand treatment caused particularly significant attenuation of cell growth (Figure 5A). CH11 also caused strong activation of caspase-8 and appearance of the p18 form in sh-NT cells, but this was again absent in sh-VDAC1 cells following CH11 treatment (Figure 5B).

Silencing of c-FLIP has been shown to induce caspase-8 activation in a number of NSCLC cell lines, including H460 [19]. Accordingly, the role of VDAC1 in regulating apoptosis induced by cFLIP knockdown was also examined. FLIP silencing by siRNA induced accumulation of caspase-8-p18 in sh-NT control cells, but this was not observed in sh-VDAC1 cells (Figure. 5C).

### Exogenous VDAC1-Myc restores TRAIL induced apoptosis in cells with silenced endogenous VDAC1

To determine whether re-expression of exogenous VDAC1 could restore sensitivity to TRAIL in cells in which endogenous VDAC1 had been selectively silenced by siRNA targeting the 3' untranslated region (3'UTR) of VDAC1, H460 clones stably expressing the full length coding region of VDAC1 fused to Myc-tag were generated. Clones 5A and 5B exhibited moderate increases in overall expression of VDAC1 compared to empty vector control cells (Figure 5E). Cells stably expressing empty vector (EV) or VDAC1-myc were treated with NT siRNA or a VDAC1 3'UTR siRNA. Following treatment with TRAIL, VDAC1-myc transfected cells exhibited increased activation of caspase-3 compared to control cells (Figure 5D). Control cells also showed a lack of TRAIL induced caspase-8 processing to p18 (Figure 5F). In VDAC1-myc overexpressing cells, however, PARP processing occurred to a greater degree compared to control (Figure 5G), and was unaffected by silencing of endogenous VDAC1.

## Discussion

The role of VDAC1 in regulating apoptosis has been the subject of considerable debate. Knockout of all three isoforms of VDAC was shown to have no effect on mitochondrial apoptosis in mouse embryonic fibrosis [5], whereas conflicting data has indicated that the N-terminal of VDAC1 is essential for release of cytochrome C following various apoptotic stimuli [22]. VDAC2 has been shown to inhibit BAK activation in a similar manner to a BCL2 family protein [2]; however recent data suggests that VDAC2 also has a proapoptotic role associated with regulation of BAK and BAX [23,24]. VDAC1 promotes aerobic glycolysis in cell lines through its interaction with hexokinase at the outer mitochondrial membrane [25,26]. Other studies have shown that hexokinase binds to mitochondria via the N-term of VDAC1 and that this is associated with resistance to mitochondrial apoptosis [27,28]. These reported interactions between VDAC1 and hexokinase imply a pro-survival rather than pro-apoptotic role for VDAC1 activity in cancerous cells.

VDAC1 interacts directly with, and may be functionally regulated by the anti-apoptotic BCL-2 family protein BCL-XL [1,29-32] or the proapoptotic BH3-only proteins BAD [33] and BID [34]. The mitochondrion has been previously implicated in BAX/BAK independent regulation of extrinsic death pathway signaling. BCL-XL can inhibit cleavage of caspase-8 at the mitochondrial surface following treatment with FAS ligand [15]. Confocal microscopy showed that caspase-8 partially colocalised with a mitochondrial marker in H460 cells, and western blot indicated that pro-caspase-8 was divided between mitochondrial and cytosolic fractions. This is consistent with previous studies which have demonstrated pro-caspase-8 and p18-caspase-8 localization to the mitochondria [16-18]. We observed that TRAIL induced cleavage of caspase-8 to the active p18 protein, which was detected predominantly in the mitochondrial compartment, and that this was dependent upon expression of VDAC1 in various NSCLC cell lines.

This study is the first to implicate VDAC1 in the regulation of extrinsic pathway mediated apoptosis. Using both stable and transient knockdown of VDAC1 we have shown that death receptor dependent apoptosis relies on the expression of this outer mitochondrial membrane protein for efficient processing of caspase-8. VDAC1 did not appear to directly affect BAX/BAK dependent activation by tBID, in agreement with a recent finding that mitochondria from VDAC1(-/-) MEFs remained sensitive to tBID [23]. Accordingly, the mitochondrial VDAC1 directly regulates the extrinsic apoptotic pathway via caspase-8 and indirectly regulates the mitochondrial death pathway via BID processing, implicating a previously unknown proapoptotic, and physiological function for VDAC1.

Although our findings suggest that downstream of death ligand binding, VDAC1 acts as a facilitator of caspase-8 processing, it is not known whether VDAC1 requires cooperation with additional proteins in the outer mitochondrial membrane or is sufficient to activate caspase-8. Recent evidence has shown that caspase-8 localisation occurs at contact sites where cardiolipin, a predominantly inner mitochondrial membrane phospholipid, is exposed at the mitochondrial surface; however, the binding interactions of caspase-8 at the mitochondria remain undefined [14]. VDAC1 is also present at contact sites on the outer mitochondrial surface raising the possibility that loss of VDAC1 could in some way alter contact site architecture and the cardiolipin platform for caspase-8 docking. One argument against this from our own and previous data however, is that significant levels of procaspase-8 are present in the mitochondrial outer membrane in the absence of TRAIL. It is therefore unclear by what molecular mechanism VDAC1 may regulate caspase-8 cleavage. Caspase-8 processing to p18 may occur at the mitochondria in a VDAC1-dependent manner leading to a signal amplification loop following binding of death ligand and DISC assembly. Such an amplification loop could be reliant on active caspase-3, which accumulates in the mitochondria following etoposide-induced cell death [18].

The structural requirements for VDAC1 regulation in regulating caspase-8 activation are unknown. The E3 ligase Cullin-3 polyubiquitinates caspase-8 prior to its cleavage at the DISC following death receptor stimulation [35], and VDAC1 could play a regulatory role in this process. We cannot rule out the possibility that VDAC1 may promote caspase-8 cleavage by associating with DISC components at the plasma membrane; redox activity of VDAC1 at the plasma membrane has been shown to regulate apoptosis [7,36]. Further studies are required to delineate the domains and protein interactions which are essential for VDAC1 to regulate caspase-8 activation; such studies are ongoing and will investigate whether this function is specific to VDAC1 alone, or shared by other VDAC isoforms due to local homology in critical domains.

## Conclusions

In summary, we have shown that VDAC1 is required for activation of caspase-8 following stimulation of the extrinsic apoptosis pathway in a panel of NSCLC cells. TRAIL is currently being investigated in the clinical setting as a potential anticancer therapy; trials are ongoing in NSCLC [20,21]. Our findings suggest that expression levels of VDAC1 could be a factor predictive for tumour sensitivity to therapeutic TRAIL receptor agonists in a subset of cancers.

## Methods

### Cell Culture and Generation of Stable shRNA Expressing Clones

ATCC-NCI-H460 Non-Small Cell Lung Cancer Cells, and MOR cells, were grown in RPMI 1640 medium (PAA) with 10% fetal calf serum and penicillin/streptomycin at 37°C with 5% CO_2_. SKMES cells were grown in EMEM media. Plasmids encoding shRNA sequences to VDAC1 and Non-Targeting (NT) shRNA (SA Biosciences) were transfected into H460 NSCLC cells using FuGene6 reagent (Roche) according to manufacturer's instructions. Plasmids bore G418 antibiotic resistance selection marker and transfected cells in 12-well plate were incubated with 0.4 mg/ml G418 until discrete colonies visible to the naked eye could be selected for transferral to a fresh plate. Three rounds of selection were preformed and clones which grew steadily in the presence of antibiotic were tested for knockdown of protein of interest by western blot. Cells were cultured continuously in the presence of appropriate antibiotics, but antibiotics were removed when plating for drug treatment.

### SiRNA knockdown

Cells were transfected with 50 nM of VDAC1 on-target siRNA smartpool (Dharmacon), VDAC1_11 siRNA sequence (Qiagen) or Non-Targeting siRNA using Dharmafect-1 reagent. Following transfection, transfection media was replaced with RPMI 1640 media, cells incubated for 24 h, trypsinized and seeded for experiments described in text (5000 cells/well for 96-well plate). Endpoint was taken 24 h post TRAIL treatment and 48 h post siRNA transfection.

### Mitochondrial Isolation and Depolarisation Experiments

Cells typically 90% confluent were detached with a cell scraper into culture media, pelleted by centrifugation to include non-adherent cells, if any, and washed three times in Mitochondrial Isolation Buffer (200 mM Mannitol, 70 mM Sucrose, 1 mM EGTA, 10 mM HEPES, 0.5 mg/ml BSA, pH7.4). Mitochondria were then isolated by Dounce homogenization followed by centrifugation at 800 g for 10 minutes to remove debris and heavy membranes, then centrifugation at 10,000 g for 10 minutes to separate mitochondria from cytosolic fraction. For measurement of mitochondrial polarization, approximately 1 mg mitochondria were resuspended in 800 μl of respiration buffer (125 mM KCl, 5 mM HEPES, 1 mM EGTA, 1 mM KHP0_4_, 2.5 mM MgCl_2_, 0.4% BSA, pH 7.4) with 5 μM Rotenone. Mitochondria were then loaded with JC-1 (10 nM) (Invitrogen), incubated for 5 minutes at 37°C, and washed and centrifuged 3 times in 800 μl MRB. 50 μl of mitochondria solution was applied to each well of a 96 well plate, with final volume made to 100 μl by applying treatment as described in text. Mitochondria were incubated at 37°C and JC-1 fluorescence measured at 530 nm (green) and 580 nm (red) with excitation at 480 nm. Results were summarized as mean ± standard deviation.

### Western Blotting

25 μg of cell lysate or mitochondrial fractions were loaded into a 12% SDS-PAGE gel. Proteins were separated by electrophoresis and transferred to PVDF membrane for blocking and incubation in 5% milk with primary and HRP-conjugated secondary antibodies. Images were developed with ECL-plus (GE healthcare). Alternatively, fluorescently tagged secondary antibodies were used for analysis of Western blot on Licor Odyssey Imaging system. Primary antibodies used were; anti-VDAC1 (Abcam ab14734), anti-PARP (E-Bioscience 14-6666-92), anti-Caspase-8 (Alexis 804-242) anti-BAX (Cell Signaling #2774), anti-COX4 (Cell Signaling #4850), anti-BID (Cell Signaling #2002), anti α-tubulin (Abcam ab15246). Secondary antibodies used were; goat anti-mouse HRP (DAKO P0447), goat anti-rabbit HRP (DAKO P0448), anti-mouse Dy-Lite800 (Rockland 610-145-121), anti-rabbit Dy-Lite680 (Rockland 611-144-122).

### ATPase Viability Assay and Caspase Activity Assays

Cells were seeded at 5000/well into 96-well plates, (minimum n = 5). After 24 h cells were treated with 10 ng/ml recombinant human TRAIL (Calbiochem) or vehicle control to final volume of 150 μl. Following 24 h treatment, luminescent ATPase assay (Lonza) was carried out according to manufacturer's instructions and 1 second luminescence readings taken on a Berthold Tristar plate reader. p-values were generated using Student's t-test.

For DEVDase activity assays, Casp3/7-glo reagent (Promega) was mixed 1:1 with RPMI and 50 μl applied to cells after removal of culture media. Plates were incubated at 21°C for 30 minutes and luminescence read as before.

### Flow Cytometry

Cells were seeded at a density of 1 × 10^5^cells/well in 6-well plates. Following treatment, DNA content was evaluated by propidium iodide (PI, Sigma) staining of cells by re-suspending them in 360 μL 0.1%FCS, 10 μg/mL PI, 0.25 mg/mL RNaseA in 1 xPBS. Samples were incubated for 30 minutes prior to analysis on a FacsScalibur Flow Cytometer (Becton Dickinson), in the FL-2 channel, to determine percentage of cells with DNA content <2N. For cell surface expression of receptor proteins, cells were seeded as before, harvested, and rinsed twice in PBS/0.2% BSA/0.2% Sodium Azide. Cells were resuspended in PBS/BSA/sodium azide and incubated at 4°C for 60 minutes with PE-conjugated antibodies to DR4, DR5, or isotype control antibody. Staining intensity was measured on flow cytometer in FL-2 channel.

### Confocal Microscopy

Cells were seeded onto a glass coverslip inside a 6-well plate and fixed in 2% paraformaldehyde. Coverslips were incubated in PBS/0.1% Triton-X with 1:1000 dilutions of anti-Caspase-8 and anti-COX-4 antibodies, for 1 h at 37°C with agitation, each followed by secondary antibodies; goat anti-mouse AlexaFluor^488 ^and goat anti rabbit AlexaFluor^594 ^(Invitrogen). Slides were mounted with ProLong gold mountant (Invitrogen) and imaged on Leica SP5 confocal microscope.

## Competing interests

The authors declare that they have no competing interests.

## Authors' contributions

ADC designed and carried out experiments and wrote the manuscript. FL carried out experiments. IP assisted with generating clones. DBL assisted with experiment design and manuscript writing. DAF designed experiments, wrote the manuscript, and directed research. All authors have read and approved the manuscript.

## Pre-publication history

The pre-publication history for this paper can be accessed here:

http://www.biomedcentral.com/1471-2407/10/380/prepub
